# Computational pathology of pre-treatment biopsies identifies lymphocyte density as a predictor of response to neoadjuvant chemotherapy in breast cancer

**DOI:** 10.1186/s13058-016-0682-8

**Published:** 2016-02-16

**Authors:** H. Raza Ali, Aliakbar Dariush, Elena Provenzano, Helen Bardwell, Jean E. Abraham, Mahesh Iddawela, Anne-Laure Vallier, Louise Hiller, Janet. A. Dunn, Sarah J. Bowden, Tamas Hickish, Karen McAdam, Stephen Houston, Mike J. Irwin, Paul D. P. Pharoah, James D. Brenton, Nicholas A. Walton, Helena M. Earl, Carlos Caldas

**Affiliations:** Cancer Research UK Cambridge Institute, University of Cambridge, Li Ka Shing Centre, Cambridge, UK; Department of Pathology, University of Cambridge, Cambridge, UK; Institute of Astronomy, University of Cambridge, Cambridge, UK; Department of Oncology, University of Cambridge, Addenbrooke’s Hospital, Cambridge, UK; Department of Histopathology, Addenbrooke’s Hospital, Cambridge University Hospitals NHS Foundation Trust, Cambridge, UK; Cambridge Experimental Cancer Medicine Centre and NIHR Cambridge Biomedical Research Centre, Cambridge, UK; Warwick Clinical Trials Unit, University of Warwick, Coventry, UK; Cancer Research UK Clinical Trials Unit, Institute for Cancer Studies, The University of Birmingham, Edgbaston, Birmingham, UK; Royal Bournemouth Hospital and Bournemouth University, Castle Lane East, Bournemouth, UK; Peterborough and Stamford Hospitals NHS Foundation Trust and Cambridge University Hospital NHS Foundation Trust, Peterborough, UK; Royal Surrey County Hospital NHS Foundation Trust, Egerton Road, Guildford, UK; Present address: Department of Anatomy and Developmental Biology, Monash University, Clayton, Victoria Australia

**Keywords:** Breast cancer, Computational pathology, Neoadjuvant, Lymphocytes, Treatment resistance, Immunology

## Abstract

**Background:**

There is a need to improve prediction of response to chemotherapy in breast cancer in order to improve clinical management and this may be achieved by harnessing computational metrics of tissue pathology. We investigated the association between quantitative image metrics derived from computational analysis of digital pathology slides and response to chemotherapy in women with breast cancer who received neoadjuvant chemotherapy.

**Methods:**

We digitised tissue sections of both diagnostic and surgical samples of breast tumours from 768 patients enrolled in the Neo-tAnGo randomized controlled trial. We subjected digital images to systematic analysis optimised for detection of single cells. Machine-learning methods were used to classify cells as cancer, stromal or lymphocyte and we computed estimates of absolute numbers, relative fractions and cell densities using these data. Pathological complete response (pCR), a histological indicator of chemotherapy response, was the primary endpoint. Fifteen image metrics were tested for their association with pCR using univariate and multivariate logistic regression.

**Results:**

Median lymphocyte density proved most strongly associated with pCR on univariate analysis (OR 4.46, 95 % CI 2.34-8.50, p < 0.0001; observations = 614) and on multivariate analysis (OR 2.42, 95 % CI 1.08-5.40, p = 0.03; observations = 406) after adjustment for clinical factors. Further exploratory analyses revealed that in approximately one quarter of cases there was an increase in lymphocyte density in the tumour removed at surgery compared to diagnostic biopsies. A reduction in lymphocyte density at surgery was strongly associated with pCR (OR 0.28, 95 % CI 0.17-0.47, p < 0.0001; observations = 553).

**Conclusions:**

A data-driven analysis of computational pathology reveals lymphocyte density as an independent predictor of pCR. Paradoxically an increase in lymphocyte density, following exposure to chemotherapy, is associated with a lack of pCR. Computational pathology can provide objective, quantitative and reproducible tissue metrics and represents a viable means of outcome prediction in breast cancer.

**Trial registration:**

ClinicalTrials.gov NCT00070278; 03/10/2003

**Electronic supplementary material:**

The online version of this article (doi:10.1186/s13058-016-0682-8) contains supplementary material, which is available to authorized users.

## Background

Women with high-risk early breast cancer are increasingly being offered chemotherapy before definitive surgery because neoadjuvant chemotherapy can enable breast-conserving surgery [1]. Complete eradication of tumour cells in the surgically removed tumour bed or pathological complete response (pCR) is associated with improved survival [2, 3]. The likelihood of pCR is profoundly affected by the oestrogen receptor (ER) and human epidermal growth factor receptor 2 (HER2) status of the primary tumour [2, 4]. In spite of these differences, improved prediction of the probability of pCR is needed because alternative regimens of chemotherapy or enrolment in clinical trials might be offered to patients deemed unlikely to experience pCR at baseline.

Novel pathological and genomic predictors of pCR have been described. Hatzis et al*.* used gene-expression microarrays to generate a gene set encompassing modules for response to endocrine therapy and cytotoxic chemotherapy, which identified patients likely to undergo pCR and to have longer survival [5]. The proportion of tumour infiltrating lymphocytes has also been shown to predict pCR in several studies of neoadjuvant chemotherapy [6–12]. Automated quantitative estimates of tumour morphology using digital images of tissue sections have been shown to be associated with prognosis [13, 14]. Therefore, comparable computational analysis of histological sections might provide a similar method for prediction of pCR.

We hypothesized that systematic quantitative analysis of tumour morphology at diagnosis would objectively identify tissue characteristics associated with pCR. We undertook a digital pathology study using a newly developed image processing method for single cell detection and material from the Neo-tAnGo randomized controlled trial [15], both from diagnosis and at surgery, in order to objectively identify tissue features associated with pCR and to investigate changes in quantitative morphological metrics between pre-treatment and post-treatment samples and their relationship to pCR.

## Methods

### Patients and clinical samples

Neo-tAnGo was a phase III, randomized trial with two-by-two factorial design addressing both the role of gemcitabine in a sequential neoadjuvant chemotherapy regimen of epirubicin/cyclophosphamide and paclitaxel, and the role of sequencing of these treatment components [15]. The trial recruited women with high-risk early breast cancer between 2005 and 2007 across 57 centres in the UK. Women with HER2-positive disease did not receive neo-adjuvant trastuzumab although most did receive adjuvant trastuzumab depending on local protocols. A total of 812 patients were included in the primary endpoint analysis [15]. Pathological complete response, the primary endpoint, was defined as the complete absence of tumour cells in resected breast tissue and axillary lymph nodes. Whether pCR had occurred was determined by independent analysis of histopathology reports by two investigators (EP and HME) as previously described [16]. The trial found no effect of the addition of gemcitabine on the proportion of cases with pCR but did find that sequencing of taxanes before anthracyclines led to an overall increase in pCR [15]. Details of eligibility and ascertainment of clinical characteristics are provided in the main trial report [15]. The trial was approved by the multicentre research ethics committee and subsequently by the local research ethics committees at all participating centres (full names and details are provided in Additional file 1). All patients provided written informed consent and the trial was registered (ClinicalTrials.gov NCT00070278).

### Image acquisition, processing and pathology review

**Fig. 1:**
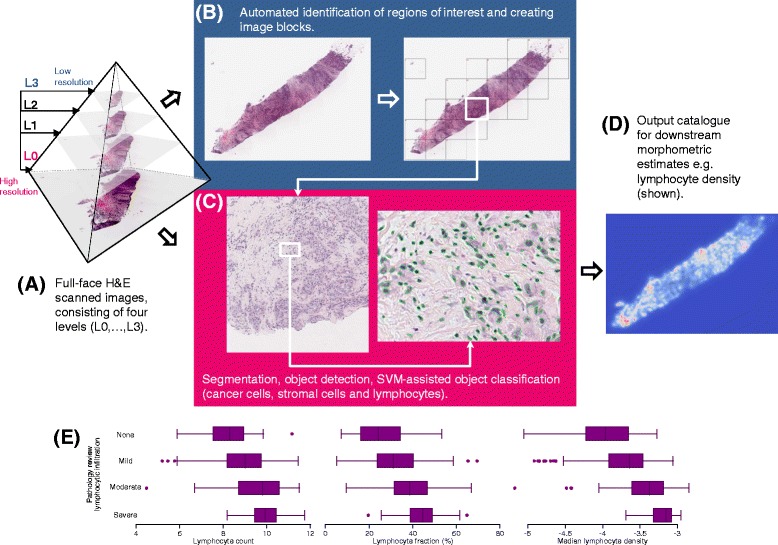
Overview of the image processing method. **a** Full-face H&E scanned images consist of four levels (L0–L3) across a gradation of resolutions. The levels L3 (lowest resolution) and L0 (highest resolution) are used to process each image. **b** Automated identification of regions of interest was performed by dividing image layer L3 into several small blocks (*grid*) and by analysing the pixel intensity distribution of each block. **c** Each image block found to contain tissue was mapped onto layer L0 and image segmentation and object detection (*green ellipses*) was conducted to construct an object catalogue. **d** Illustrative representation as a contour map of lymphocyte density derived using a k-nearest neighbour algorithm of the 50 nearest like-class neighbours. **e** Distribution of lymphocyte metrics by categories of lymphocytic infiltration based on central pathology review. *SVM* support vector machine

Haematoxylin and eosin (H&E)-stained histological slides from formalin-fixed paraffin embedded (FFPE) pre-treatment core biopsies and tumours resected at surgery were requested from all centres for central review and digitization. Image analysis was conducted by AD at the Institute of Astronomy in Cambridge, as part of a collaboration with Oncology [17]. Our image-processing pipeline is summarized in Fig. 1. Our approach is entirely automated and consists of identifying tissue for analysis, segmenting cell nuclei and finally using machine-learning to classify nuclei as cancer, stromal or lymphocyte based on a training set. Adipocytes were included in the stromal category because based on nuclear features alone, it was not possible to reliably identify them. Further details are provided in Additional file 1.

### Statistical analyses

All image analyses were conducted prior to receipt of clinical data from the trial statistician. Associations between continuous automated image metrics and categorical clinical variables were tested using Wilcoxon’s rank-sum test or the Kruskal-Wallis test. Pearson’s correlation coefficient was used to investigate the relationship between continuous variables. Logistic regression was used to test for associations with pCR providing an odds ratio (OR), 95 % confidence interval (CI) and *p* value. Candidate image-based predictors were initially tested against pCR in univariate analysis, with absolute cell counts as log-transformed variables. Next, a multivariate model was fitted iteratively in a backward stepwise manner to retain metrics significantly associated with pCR after adjustment for others remaining in the model. These metrics were finally included in a multivariate analysis further adjusted for patient age, radiological tumour size at diagnosis, lymph node status, histological grade, ER status and HER2 status. All image metrics were modelled as continuous variables. Whether an image metric predicted pCR differently between taxane sequencing groups (first vs second) was investigated by inclusion of an interaction term in a logistic regression model and the fit of this model compared to a model lacking the term using a likelihood ratio test. Statistical analyses were conducted in Intercooled Stata version 11.2 (Stata Corp, College Station, TX, USA). This study was conducted in compliance with the Reporting recommendations for tumor marker prognostic studies (REMARK) criteria [18] as detailed in Additional file 1.

## Results

### A digital pathology resource and image-processing pipeline for quantitative cell-level metrics

We used material from 765 patients enrolled in the Neo-tAnGo randomized controlled trial. Additional file 2 depicts the flow of patients through each analytical stage in this study. A total of 2,436 slides were received and digitized, of which 1,992 contained tumour or tumour bed with the remainder containing adjacent normal breast tissue. All images used for analyses are available to download together with open-source image analysis code at http://www.ast.cam.ac.uk/~adariush/files/images/ and http://www.ast.cam.ac.uk/~adariush/files/codes/.

On average, each patient’s tumour or residual tumour bed was represented in 1.2 pre-treatment slides (range 1–23) and 1.8 slides from the post-treatment surgical specimen (range 1–35). Figure 1 summarizes our novel image processing pipeline: scanned slide images were extracted as four layers differing in their size and resolution, and using the lowest resolution image, areas that contained tissue were automatically identified and white space was excluded from further analysis. Next, using the highest resolution layer, single cell nuclei were detected, and using a support-vector-machine (SVM) approach trained using around 1,000 objects per class, cells were sub-classified into readily distinguishable categories (cancer, stromal or lymphocyte). Metrics relating to these three cell types and describing absolute cell number (count), relative cell type proportion (fraction) and density (minimum, median and maximum) based on the 50 nearest like-class neighbors, were computed. That is, every detected cell was assigned a density estimate based on the distance between it and the 50 nearest cells of the same type. Pathologist assessment of lymphocytic infiltration in pre-treatment biopsies with paired automated data, to which the pathologist was blinded, was available for comparison in 377 samples. All automated metrics of lymphocytic infiltration were significantly associated with pathologist scores (*p* <0.0001 for all three comparisons; Fig. 1), attesting to the validity of the automated image analysis approach.

Image metrics were calculated for a total of 765 patients of which 623 provided data on pre-treatment biopsies and 699 provided data on post-treatment surgical samples, with paired (pre- and post-treatment) data available in 557 patients. Patient and tumor characteristics are detailed in Table 1. Fifteen machine-learning-derived image metrics were used for downstream correlative analysis with clinical and pathological features. Additional files 3 and 4 depict histograms of the distributions of image metrics for pre-treatment biopsies and post-treatment surgical samples. The correlation matrix of image metrics from data generated using pre-treatment biopsies is depicted in Additional file 5 and from surgical samples in Additional file 6. In pre-treatment biopsies, the strongest positive correlation was between the absolute number of lymphocytes and the number of cancer cells (rho = 0.84) and, conversely, the strongest negative correlation was between the relative fraction of cancer cells and the fraction of lymphocytes (rho = –0.59). These contrasting correlations highlight the extent to which absolute cell counts simply reflect sample cellularity, warranting cautious interpretation.

**Table 1:** Patient and tumour characteristics

	Number	Percent
Tumour size		
≤50 mm	613	80.1
>50 mm	152	19.9
Total	765	100
Node status		
Negative	388	50.7
Positive	377	49.3
Total	765	100
Grade		
1	22	2.9
2	245	32
3	328	42.9
Missing	170	22.2
Total	765	100
Taxane sequence		
Taxane first	387	50.6
Taxane second	378	49.4
Total	765	100
pCR		
No pCR	633	82.7
pCR	122	15.9
Missing	10	1.3
Total	765	100
ER, HER2 status		
ER–, HER2–	152	19.9
ER–, HER2+	64	8.4
ER+, HER2–	342	44.7
ER+, HER2+	116	15.2
Missing	91	11.9
Total	765	100
Diagnostic biopsies		
Missing	142	18.6
Analysed	623	81.4
Total	765	100
Surgical samples		
Missing	66	8.6
Analysed	699	91.4
Total	765	100

*pCR* pathological complete response, *ER* oestrogen receptor, *HER2* human epidermal growth factor receptor 2

### Image metrics reflect molecular subtype of the primary tumor

**Fig. 2:**
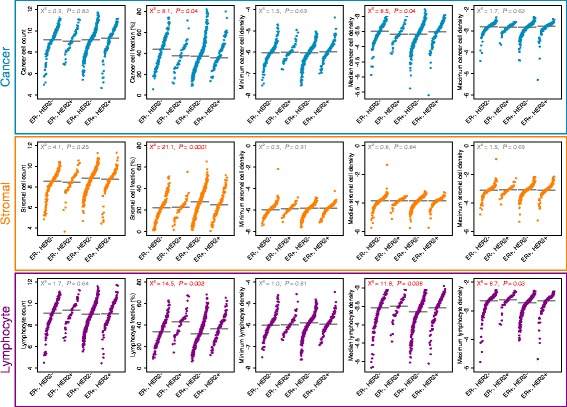
Distribution of pre-treatment sample image metrics by tumour molecular subtype. *Horizontal grey lines* represent median values. Results of the Kruskal-Wallis test are depicted within graphs; *red text* denotes *p* values <0.05. *ER* oestrogen receptor, *HER2* human epidermal growth factor receptor 2

Figure 2 depicts the distribution of image metrics by the molecular subtype of the tumour based on ER and HER2 status. The distribution of cancer cell fraction significantly differed between groups (*p* = 0.04), with the highest median cancer cell fraction observed in ER-negative, HER2-negative tumours. Similarly, the distribution of stromal cell fraction was significantly different between groups (*p* = 0.00001), with the highest median level observed in ER-positive, HER2-negative tumours. The distribution of lymphocyte fraction was also significantly different between groups (*p* = 0.002) with the highest median level observed in ER-negative, HER2-positive tumours. Median lymphocyte density was not correlated with patient age at diagnosis (Pearson’s correlation coefficient –0.01), suggesting that the association with tumour molecular subtype was not age-related. However, these relationships did not hold following chemotherapy in post-treatment surgical samples for which none of the metrics were significantly different across molecular subtypes (Additional file 7), likely due to the diminishing influence of tumour cells on the composition of samples, owing both to widespread cell death and to other effects of chemotherapy on the tissue environment.

### Chemotherapy response is most strongly associated with median lymphocyte density

**Fig. 3:**
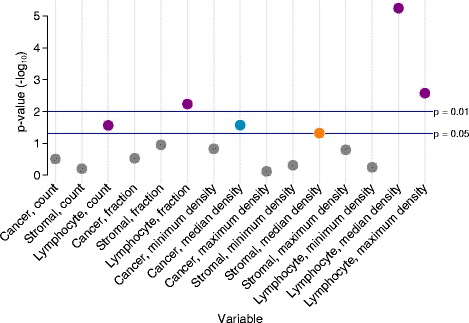
Association between image metrics and pathological complete response (pCR). Manhattan plot illustrates *p* values (–log10) from univariate logistic regression analyses testing the association between 15 image metrics and pCR

Univariate analysis of the 15 image metrics was conducted using pretreatment biopsies in 614 patients, of whom 98 (16 %) had tumors that underwent pCR. Six of the fifteen metrics were significantly (at a nominal *p* value <0.05) associated with pCR (Fig. 3 and Additional file 8). Of these six, four were related to lymphocytes, one to cancer cells and one to stromal cells. The association between median lymphocyte density and pCR was by far the strongest (OR 4.46, 95 % CI 2.34-8.50, *p* <0.0001) with the next most significant being maximum lymphocyte density (OR 3.48, 95 % CI 1.54, 7.86, *p* = 0.003). To determine whether sample cellularity influenced the relationship between median lymphocyte density and pCR, a model adjusted for the total number of cells in a sample was fit. This showed that sample cellularity had little effect on this association, which remained significant in the model (OR 4.56, 95 % CI 2.27, 9.15, *p* <0.0001). A multivariate model comprising these 15 predictors was modified in a backward stepwise manner resulting in a final model of 5 significant predictors, including median lymphocyte density (Additional file 8). However, on further adjustment of this model for clinical variables only median lymphocyte density was significantly associated with pCR (Additional file 9). When included in a multivariate model with only clinical predictors, median lymphocyte density remained significantly associated with pCR (OR 2.42, 95 % CI 1.08, 5.40, *p* = 0.03; Table 2). However, approximately one third of observations were lost between univariate (n = 614) and multivariate analyses (n = 406). For deciles of median lymphocyte density, differences in the proportion of cases that underwent pCR varied between 4.9 % (3/61) for the first decile up to 35.5 % (22/62) for the last.

**Table 2:** Univariate and multivariate logistic regression models of clinical factors and median lymphocyte density

		Univariate				Multivariate			
Variable	Categories	Odds ratio	95 % CI	*P* value	Observations	Odds ratio	95 % CI	*P* value	Observations
Age	Continuous	0.99	0.97, 1.02	0.6	755	0.99	0.96-1.02	0.44	406
Tumour size	≤50 mm, >50 mm	0.89	0.54, 1.48	0.66	755	0.68	0.27-1.75	0.43	406
Node status	Negative, positive	0.8	0.54, 1.18	0.25	755	0.56	0.31-1.00	0.05	406
Grade	1, 2, 3	3.98	2.38, 6.67	<0.00001	588	3.62	1.74-7.52	0.0006	406
ER status	Negative, positive	0.26	0.17, 0.38	<0.00001	755	0.3	0.17-0.53	0.00004	406
HER2 status	Negative, positive	1.6	1.03, 2.50	0.04	665	1.81	0.97-3.37	0.06	406
Median lymphocyte density	Continuous	4.46	2.34, 8.50	<0.00001	614	2.42	1.08-5.40	0.03	406

*ER* oestrogen receptor; *HER2* human epidermal growth factor receptor 2

**Fig. 4:**
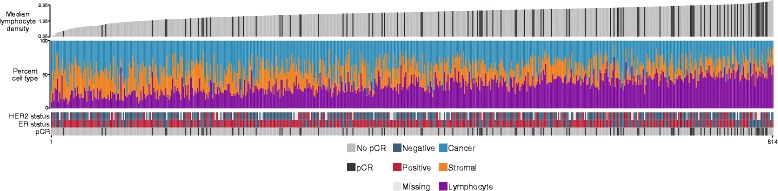
Relationship between median lymphocyte density, cellular composition, pathological complete response (*pCR*) and receptor status. Bar plots illustrating the relationship between pathological complete response, oestrogen receptor (*ER*) status, human epidermal growth factor receptor 2 (*HER2*) status, cellular composition and median lymphocyte density. Plots are sorted by increasing level of median lymphocyte density. For illustration, median lymphocyte density has been rescaled to positive values

Figure 4 depicts the distribution of pCR, receptor status and cellular composition in all samples ranked according to the median lymphocyte density for each sample. In addition to depicting the sample-level relationship between these variables, the relationship between median lymphocyte density, pCR and molecular subtype is also depicted in Fig. 4. To address whether the association between median lymphocyte density and pCR significantly differed by ER status, we compared the fit of two logistic regression models: one with an interaction term between median lymphocyte density and ER status, and one without. The likelihood ratio test comparing these models was not significant (*p* = 0.72), however, it should be noted that for comparison of effect between subgroups these analyses are relatively underpowered, precluding reliable conclusions. In addition, Fig. 4 highlights that although higher median lymphocyte density is generally associated with a larger lymphocyte fraction (rho = 0.69), it is not simply a reflection of higher numbers of infiltrating lymphocytes. There are both instances where the relative fraction of lymphocytes is high but where the density of lymphocytes is low compared to other samples, and conversely in some instances the fraction of lymphocytes is relatively low but their median density is high (Fig. 4). Additional file 10 depicts this relationship as a scatter plot, further highlighting the existence of outlier cases. This suggests that a measure of lymphocytic density may reflect a functional aspect of the immune response not entirely encompassed by lymphocyte fraction. Additional file 11 depicts example images together with contour representations of lymphocyte density and automated metrics for each image. Median lymphocyte density was positively associated with both axillary lymph node status (*p* = 0.002) and histological grade (*p* <0.00001) but not with tumor size (*p* = 0.47) as depicted in Additional file 12.

### Increased post-treatment lymphocyte density is associated with relative chemoresistance

**Fig. 5:**
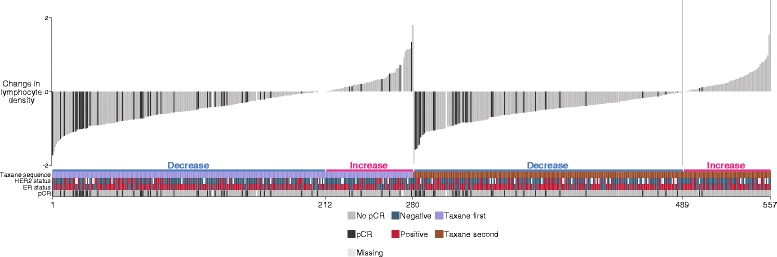
Change in lymphocyte density and association with pathological complete response (*pCR*). Plot depicts the change in median lymphocyte density between paired pre-treatment and post-treatment surgical samples. The primary sort key is taxane sequence and the secondary sort key is change in lymphocyte density. Annotated rug depicts oestrogen receptor (*ER*), human epidermal growth factor receptor 2 (*HER2*) status and taxane sequence for samples corresponding to those depicted in the plot above

We further investigated whether the change in median lymphocyte density between pre- and post-treatment samples was reflected in the probability of pCR and the extent to which the degree of change varied between patients. Change in median lymphocyte density across all 557 samples for which paired data was available is depicted as a waterfall plot in Fig. 5. In the majority of cases (75.6 %, 421/557 cases) lymphocyte density decreased in the surgical specimen. In addition, on assessment of the association between change in lymphocyte density and pCR in the 553 cases with sufficient data, there was strong association between reduction in lymphocyte density in the surgical sample and higher likelihood of pCR (OR 0.28, 95 % CI 0.17, 0.47, *p* <0.0001) with 17 % (71/418) of cases with a decrease in density undergoing pCR, in comparison to just 6.7 % (9/135) of those where there was an increase. Change in lymphocyte density was negatively correlated with median lymphocyte density at diagnosis (Correlation coefficient –0.6, *p* <0.0001). In a model adjusted for median lymphocyte density at diagnosis, change in lymphocyte density remained significantly associated with pCR (OR 0.38, 95 % CI 0.21, 0.71, *p* = 0.002).

The main finding of the Neo-tAnGo trial was that administration of a taxane prior to other chemotherapy agents led to a significant increase in pCR [15]. Therefore, we next evaluated whether the association between increased post-treatment lymphocyte density and reduced likelihood of pCR was equally distributed according to whether a taxane was received first or second (Fig. 5). The association between increased post-treatment lymphocyte density and reduced likelihood of pCR was significantly stronger where a taxane was received second compared to where it was received first (likelihood-ratio test for interaction, *p* = 0.02). The OR in the taxane second group was 0.12 (95 % CI 0.05, 0.31, *p* <0.0001) compared to 0.45 (95 % CI 0.24, 0.84, *p* = 0.01) in the taxane first group. It should be noted that the proportion of cases with increased post-treatment lymphocyte density was equally distributed across taxane groups (24 % and 25 %).

## Discussion

We used computational pathology to generate image metrics of both pre- and post-treatment biopsies in a randomized controlled trial of neoadjuvant chemotherapy in breast cancer in order to investigate associations with chemosensitivity. Median lymphocyte density in pre-treatment biopsies emerged as the best predictor of response to chemotherapy, improving prediction based on known clinical factors. In addition, change in lymphocyte density between pre and post-treatment samples revealed that an increase in lymphocyte density was, paradoxically, associated with relative chemoresistance.

Computational pathology was used here to generate objective quantitative estimates of the cellular composition of tissue samples and, importantly, of the spatial heterogeneity of different cell types across a tissue section. Median density of lymphocytes, a spatial estimate, outperformed simple cellular quantification. Similarly, we have previously reported that the spatial distribution of stromal cells is prognostic in breast cancer and that this feature is not easily measurable by genomic assays [13]. Previous work in the context of breast and prostate cancer, has demonstrated the capacity of a computational approach to interrogate spatial, relational and geometric features of tissues for outcome prediction [14, 19, 20]. Many of these parameters could not be practically estimated by other means, and in this respect machine learning can provide deeper insight into tissue morphology than is possible by manual evaluation.

We performed a data-driven selection of tissue features associated with pCR. Median lymphocyte density emerged as the best predictor of chemosensitivity. In a previous study we have reported an association between automated estimates of lymphocytic infiltration and breast cancer survival in ER-negative disease [13, 21]. Similarly, studies of patients who received neo-adjuvant chemotherapy based on genomic assays and histopathology have also reported an association between the immune response and pCR [22]. Ignatiadis et al*.* conducted a meta-analysis of gene-expression data from pre-surgical specimens in 996 patients and investigated associations between 17 previously reported gene modules and pCR [22]. They found that the gene modules most reliably associated with pCR across cancer subtypes were those relating to the immune response. Tumour infiltrating lymphocytes estimated by a pathologist from H&E sections have also been found to be associated with outcome and response to chemotherapy [8, 23], including some in the neo-adjuvant setting [6, 10], largely in accord with the findings of this study.

To our knowledge, this is the first report showing that an increase in lymphocyte density following the perturbation of chemotherapy is associated with a lower likelihood of pCR. Previous studies have also shown that the composition of the post-treatment immune repertoire is associated with survival [24, 25]. In addition, we found that the sequence in which chemotherapy agents were administered affected the strength of this association. Where patients received a taxane second, increased lymphocyte density was more strongly associated with relative resistance to chemotherapy than in patients who received a taxane first.

A limitation of this study is that we were not able to digitize and analyse samples from all patients enrolled in the Neo-tAnGo trial and that associated clinical data were not complete, leading to a loss of around one third of observations in multivariate analyses. This is inevitable in the context of large multicentre trials. A second limitation is that by using H&E sections we have not accounted for the immune phenotype or functional state of infiltrating lymphocytes.

The functional basis of the interaction we observed between the immune response and chemotherapy is uncertain. It should, however, be noted that all patients received an anthracycline (epirubicin) as part of their treatment. Anthracyclines have been extensively investigated in pre-clinical studies as a chemotherapeutic agent with a tumoricidal effect that can in part be attributed to stimulation of the immune response [26]. For example, a recent study reported that upon exposure to an anthracycline tumour cells produce type I interferons evoking an immunological cascade reminiscent of that seen in cells infected by a virus and that this effect may be partly explained by the release of self-RNAs by dying cells [27]. That greater clinical benefit of anthracyclines is significantly associated with the presence of a pre-existing immune response (tumour-infiltrating lymphocytes) has also been shown in several clinical studies [8, 23, 28]. However, our results suggest that the effect of chemotherapy may be more complex than simply boosting pre-existing immune attack. First, we find that where the density of lymphocytes is increased following treatment, fewer tumours undergo pCR. That is, in this subset of around one quarter of patients, tumour cells apparently continue to resist the effects of immune attack in spite of its increased intensity. Second, we find that this effect is significantly greater where a taxane (paclitaxel) was administered after, as opposed to before, other agents. In the Neo-tAnGo trial, giving paclitaxel before the other agents significantly increased the proportion of cases with pCR [15]. While the association between increased post-treatment lymphocyte density and treatment resistance holds whether paclitaxel is given first or second, the effect is significantly larger in cases where it is given second. Collectively, these findings raise the possibility that not only is a chemotherapy-stimulated immune response not universally effective, but that the efficacy of this response can be influenced by the sequence in which tumour cells are exposed to different chemotherapeutic agents, most notably taxanes and anthracyclines.

The existence of a substantial subgroup of relatively resistant tumours in which lymphocyte density is increased following chemotherapy further suggests a clinical opportunity. The variability in the immune response between primary breast tumours is well-known and recent genomic analyses suggest that some of this difference may be explained by the mutational burden of the primary tumour [29]. Analyses of clinical trials of immune checkpoint inhibitors report that responses are best where there is a significant pre-existing immune response to the primary tumour [30, 31]. Given that a large subset of breast tumours evoke only a mild immune response if any [23, 32], methods for increasing immune attack against immunologically quiescent tumours are needed. Therefore the subgroup we have identified may benefit from receiving chemotherapy first, to increase the immune response, followed by immune checkpoint inhibitors to amplify its effect.

Digitization of pathology slides from clinical trials affords the important advantages of providing an enduring archive of tumour pathology and the opportunity for systematic image analysis. We anticipate analyses such as ours becoming more common as digital pathology is implemented more widely in clinical trials. We provide a valuable resource of digital pathology images to the research community, together with all our image-analysis codes. In addition, linked multiplatform genomic annotation (gene expression, copy number, targeted sequencing) will be made available for a subset of cases, following primary reporting of the data. While invaluable resources such as the Human Protein Atlas already provide access to an enormous array of tissue images [33], some already utilized in translational studies [34, 35], widely available digital images of tumour tissue from large high-quality clinical studies with molecular annotation such as ours are currently exceedingly rare. However, general access to such resources will be necessary to fulfil the potential of computational pathology as a novel modality that spans the research and clinical arenas.

## Conclusions

This data-driven analysis of computational pathology metrics reveals that median lymphocyte density is an independent predictor of response to neoadjuvant chemotherapy in breast cancer. Paradoxically, we find that an increase in the density of lymphocytes following chemotherapy is associated with relative resistance to chemotherapy. Computational pathology is a novel and quantitative method for interrogation of tumour tissues, which can improve prediction of clinical endpoints.

## Additional files

Additional file 1:
**Supplementary methods.** (DOCX 38 kb) Additional file 2:
**CONSORT diagram illustrating the flow of patients at each analytical stage.** (PDF 7 kb) Additional file 3:
**Histograms depicting the distribution of all image metrics from pre-treatment samples.** (PDF 25 kb) Additional file 4:
**Histograms depicting the distribution of all image metrics from post-treatment surgical samples.** (PDF 26 kb) Additional file 5:
**Correlation matrix of all image metrics from pre-treatment samples.** Correlation matrix of 15 image metrics derived from pre-treatment biopsies. Correlations are Pearson’s coefficients. Text size is proportional to the strength of the correlation. *Pink boxes* denote positive correlations and *blue boxes* denote negative correlations. (PDF 82 kb) Additional file 6:
**Correlation matrix of all image metrics from post-treatment surgical samples.** Correlation matrix of 15 image metrics derived from post-treatment surgical samples. Correlations are Pearson’s coefficients. Text size is proportional to the strength of the correlation. *Pink boxes* denote positive correlations and those blue *boxes* denote negative correlations. (PDF 82 kb) Additional file 7:
**Distribution of post-treatment sample image metrics by tumor molecular subtype.** *Horizontal gray lines* represent median values. Results of Kruskal-Wallis tests are depicted within graphs; *red text* denotes *p* values <0.05. (PDF 177 kb) Additional file 8:
**Univariate and multivariate logistic regression models for all 15 image metrics.** (DOCX 16 kb) Additional file 9:
**Multivariate logistic regression model including clinical prognostic factors and image metrics derived from model depicted in Additional File 8.** (DOCX 14 kb) Additional file 10:
**Scatter plot of median lymphocyte density and lymphocyte fraction in pre-treatment biopsies.** Depicted rho value is a Pearson’s correlation coefficient. *a.u.* arbitrary units. (PDF 19 kb) Additional file 11:
**Examples of images and associated metrics from cases with varying median lymphocyte density.** Depicted density metrics have been arbitrarily offset to depict positive values but are normalized across samples, hence comparable between plots (*right y-axis*). Note that absolute counts are depicted against different scales (*left y-axis*). (PDF 63 kb) Additional file 12:
**Distribution of median lymphocyte density from pre-treatment biopsies by clinical variables.** *Horizontal gray lines* represent median values. Results of Kruskal-Wallis tests are depicted within graphs; *red text* denotes *p* values <0.05. (PDF 62 kb)

## Abbreviations

CI: confidence interval
ER: oestrogen receptor
FFPE: formalin-fixed paraffin-embedded
H&E: haematoxylin and eosin
HER2: human epidermal growth factor receptor 2
OR: odds ratio
pCR: pathological complete response
SVM: support vector machine
