# Supplementary material for: Computational pathology of pre-treatment biopsies identifies lymphocyte density as a predictor of response to neoadjuvant chemotherapy in breast cancer
Source: Breast Cancer Res. 2016 Feb 16;18:21. doi: 10.1186/s13058-016-0682-8 (PMC4755003; doi:10.1186/s13058-016-0682-8)
Supplement: Additional file 1: — Supplementary methods. (DOCX 38 kb) [file 13058_2016_682_MOESM1_ESM.docx]

**Supplementary Methods**

**Ethical Approval**

Neo-tAnGo was granted Clinical Trials Authorisation from the Medicines and Healthcare products Regulatory Agency (MHRA) on June 17, 2004, and approved by the multicentre research ethics committee nationally on Nov 1, 2004. The study was undertaken by the UK National Cancer Research Institute (NCRI). Research Ethics Committee (REC) Reference Number 04/MRE01/60: Approved - Nov 1, 2004; Research Ethics Committee: South-East Multi-Centre Ethics Committee, Kent and Medway Strategic Health Authority, Preston Hall, Aylesford, Kent, ME20 7NJ. The Institutional Review Boards (IRBs) at the following centres in the UK subsequently approved the trial: Addenbrooke's Hospital, Royal Surrey County Hospital, Peterborough District Hospital, Royal Bournemouth Hospital, Guy's Hospital, Nottingham City Hospital, Christie Hospital, Poole Hospital, Charing Cross Hospital, St Bartholomew's Hospital, West Middlesex University Hospital, Princess Royal Hospital, Crosshouse Hospital, St James's University Hospital, Cheltenham General Hospital, Royal Lancaster Infirmary, Dorset County Hospital, Leicester Royal Infirmary, Royal Devon and Exeter Hospital, Southend Hospital, Southport and Formby District General Hospital, Weston Park Hospital, Falkirk Royal Infirmary, Queen's Hospital, New Cross Hospital, North Middlesex Hospital, St Mary's Hospital, Aberdeen Royal Infirmary, Western Infirmary, Ayr Hospital, Burnley General Hospital, Essex County Hospital, Furness General Hospital, Southampton General Hospital, St Margaret's Hospital, The James Cook University Hospital, Clatterbridge Centre for Oncology, Cookridge Hospital, Hairmyres Hospital, Whipps Cross University Hospital, Worcester Royal Hospital, Derbyshire Royal Infirmary, Royal Free Hospital, Royal Liverpool University Hospital, Whiston Hospital, Glan Clwyd Hospital, Newham University Hospital, Gloucester Royal Hospital, Torbay Hospital, University College London Hospital, University Hospital Of North Tees, West Suffolk Hospital, Whittington Hospital, Huddersfield Royal Infirmary, Royal Preston Hospital, Salisbury District Hospital and St George's Hospital.

### Image acquisition, processing and pathology review

Haematoxylin and eosin (H&E) stained histological slides from formalin-fixed paraffin embedded (FFPE) pre-treatment core biopsies and tumors resected at surgery were requested from all centers for central review and digitization. Diagnostic slides were reviewed by the study pathologist (E.P.) and scored for a variety of histological features including lymphocytic infiltration. Slides were scanned using an Aperio Scanscope XT scanner (Leica Microsystems Ltd, Milton Keynes, UK) at 20x resolution (0.5 microns per pixel) and extracted as proprietary SVS files for analysis.

Image analysis was conducted by A.D. at the Institute of Astronomy in Cambridge as part of an ongoing collaboration with Oncology^3^. Our image-processing pipeline is summarized in Figure 1. The software used for image analysis was written entirely in Python and is based on the Open Source Computer Vision Library (OpenCV: <http://opencv.org/>), a library of C/C++ programming functions designed for image processing and real-time applications, as well as OpenSlide^4^ (http://openslide.org/) libraries. The latter provides an interface to read whole-slide images, here the Aperio SVS image format, consisting of a single-file tiled TIFF with non-standard metadata and compression. Both libraries have Python interfaces and support all major operating systems.

In order to generate an object catalogue associated with each whole-slide image, the following approach was taken: A whole-slide image consists of four independent layers L0, L1, L2 and L3, sorted in descending order (from high to low) in terms of pixel resolution such that higher level images are scaled down versions of lower level images by constant factors along either axis. To begin, the L3 image was extracted, transformed into grey-scale and inverted. The image was then divided into *N* sub-blocks of width *W* and height *H* such that (i) they cover the whole L3 image area, and (ii) the area associated to each L3 sub-block on the L0 layer, always falls within the range between 1,000 x 1,000 pixels^2^ and 2,000 x 2,000 pixels^2^. To identify blocks devoid of tissue for exclusion from further analysis, histograms of pixel-intensities were analyzed to identify blocks where the majority of pixels had intensities below a given threshold.

Next for each whole-slide image, blocks associated to those already identified in L3 were extracted from the highest resolution L0 layer. Each L0 block was transformed from RGB to YCrCb color space and the inversion of the Cb channel was used as the reference for image segmentation. An algorithm for image segmentation based on adaptive thresholding of the image global histogram was applied to the block reference image in order to create a binary image of individual objects (cell nuclei). Then for each object, image moments (position, size, area etc.) were computed and a catalogue of all objects was generated.

The object catalogue generated was predominantly composed of cell nuclei but also occasional detections belonging to pen-marks or other artefacts. In order to exclude artifactual detections we applied a non-linear support-vector machine (SVM) method (accuracy>98%) on two different color parameter spaces using a radial based function (RBF) kernel. These features were used to train a support-vector machine (SVM) based classifier to identify cancer cells, stromal cells and lymphocytes based on a training set of objects selected by a pathologist (H.R.A.) of approximately 1,000 objects for each category. First, lymphocyte and non-lymphocyte objects were classified using a parameter space based on color and the object size (cell's semi-minor axis) with an estimated accuracy of 90%. Next, non-lymphocyte objects were divided into tumor or stromal categories by applying a linear SVM method to the object's color and eccentricity parameter space with an estimated accuracy of 79%, where eccentricity is defined as sqrt(1-(*b*^2/*a*^2)) where *a* and *b* are object's semi-major and semi-minor axes.

Finally, in order to estimate the density of individual cells belonging to a given class, we computed the surface density around each cell. This was done based on counting the number of nearest neighbours, i.e. the density within the distance to the *N^th^* nearest neighbour. Hence, the surface density to the *N^th^* nearest neighbour was calculated as:

Sigma_N (pixel^[-2]) = N/(pi* d_N^2)

Where d_N is the distance to the *N^th^* nearest neighbour within a density-defining population. We tested different values for *N* (25, 50, 75 and 100) but did not find any significant difference between estimates. As such, a value of *N*=50 was used in order to estimate the density parameter. An upper limit of 100 objects was chosen for initial testing to ensure density estimates remained relatively local to each object.

### Processing time based on an Intel® Core™ i5-3427U CPU at 1.80GHz (32-bit Operating system) was ~4.7 min per gigapixel (80% core image processing and 20% for object classification and statistical analysis). Object classification alone took around ten seconds.

**SUPPLEMENTARY METHODS**

**REMARK CRITERIA COMPLIANCE CHECK-LIST**

This check-list is provided in the published REMARK report (in black text)^6^. Compliance statements for this study are highlighted in blue text.

**INTRODUCTION**

1 State the marker examined, the study objectives, and any pre-specified hypotheses.

Details provided in the Introduction.

**MATERIALS AND METHODS**

Patients

2 Describe the characteristics (for example, disease stage or co-morbidities) of the study patients, including their source and inclusion and exclusion criteria.

Patients were from the Neo-tAnGo randomised controlled trial^1^ as stated in the introduction. Patient characteristics are detailed in Table 1. Inclusion and exclusion criteria were as per trial protocols detailed in the original report^1^; this study sought to analyse data from all patients enrolled in the trial.

3 Describe treatments received and how chosen (for example, randomized or rule-based).

Treatments received are described in the methods. Patients were randomised to receive one of four treatment regimens detailed in Table 1 and Methods.

Specimen characteristics

4 Describe type of biological material used (including control samples) and methods of preservation and storage.

Samples were haematoxylin and eosin stained histological slides, digitised for computational analysis. Tumour material was formalin-fixed and paraffin-embedded.

Assay methods

5 Specify the assay method used and provide (or reference) a detailed protocol, including specific reagents or kits used, quality control procedures, reproducibility assessments, quantitation methods, and scoring and reporting protocols. Specify whether and how assays were performed blinded to the study endpoint.

Histological preparation of slides was conducted locally at numerous recruiting centres following standard procedures in diagnostic histopathology laboratories. Slide digitisation and analysis procedures are detailed in the methods. Full access to images and code to reproduce the dataset used for correlation with clinical endpoints are available to researchers. All image analysis and preliminary analysis of output catalogues was conducted prior to access to clinical data being granted by the trial statistician.

Study design

6 State the method of case selection, including whether prospective or retrospective and whether stratification or matching (for example, by stage of disease or age) was used. Specify the time period from which cases were taken, the end of the follow-up period, and the median follow-up time.

Cases were prospectively selected as per trial protocols. Pathological complete response was used a surrogate clinical endpoint in this study hence details on follow-up time are not relevant.

7 Precisely define all clinical endpoints examined.

Pathological complete response is defined in the methods.

8 List all candidate variables initially examined or considered for inclusion in models.

Full details of candidate predictors (fifteen image metrics) are provided.

9 Give rationale for sample size; if the study was designed to detect a specified effect size, give the target power and effect size.

Study sample size was powered to detect differences in treatment effect detailed in the trial report^1^. We sought to enrol all patients in the original trial for this substudy.

Statistical analysis methods

10 Specify all statistical methods, including details of any variable selection procedures and other model-building issues, how model assumptions were verified, and how missing data were handled.

Details are provided in the methods section. Missing data were handled by complete case analysis.

11 Clarify how marker values were handled in the analyses; if relevant, describe methods used for cutpoint determination.

Details are provided in the methods section. No cut-points were applied for correlative analyses.

**RESULTS**

Data

12 Describe the flow of patients through the study, including the number of patients included in each stage of the analysis (a diagram may be helpful) and reasons for dropout. Specifically, both overall and for each subgroup extensively examined report the number of patients and the number of events.

This is detailed in a flow diagram (Supplementary Figure) and within the main results section.

13 Report distributions of basic demographic characteristics (at least age and sex), standard (disease-specific) prognostic variables, and tumor marker, including numbers of missing values.

Details are provided in Table 1.

Analysis and presentation

14 Show the relation of the marker to standard prognostic variables.

Provided in Figures 1, 3 and Supplementary Figure.

15 Present univariable analyses showing the relation between the marker and outcome, with the estimated effect (for example, hazard ratio and survival probability). Preferably provide similar analyses for all other variables being analyzed. For the effect of a tumor marker on a time-to event outcome, a Kaplan-Meier plot is recommended.

Details are provided in Supplementary Table 1.

16 For key multivariable analyses, report estimated effects (for example, hazard ratio) with confidence intervals for the marker and, at least for the final model, all other variables in the model.

Provided in Table 2 and Supplementary Tables 1 and 2.

17 Among reported results, provide estimated effects with confidence intervals from an analysis in which the marker and standard prognostic variables are included, regardless of their statistical significance.

Provided in Table 2.

18 If done, report results of further investigations, such as checking assumptions, sensitivity analyses, and internal validation.

**DISCUSSION**

19 Interpret the results in the context of the pre-specified hypotheses and other relevant studies; include a discussion of limitations of the study.

Other studies and limitations are discussed.

20 Discuss implications for future research and clinical value.

These are discussed.

**References**

1. Earl HM, Vallier AL, Hiller L, et al: Effects of the addition of gemcitabine, and paclitaxel-first sequencing, in neoadjuvant sequential epirubicin, cyclophosphamide, and paclitaxel for women with high-risk early breast cancer (Neo-tAnGo): an open-label, 2x2 factorial randomised phase 3 trial. Lancet Oncol 15:201-12, 2014

2. Provenzano E, Vallier AL, Champ R, et al: A central review of histopathology reports after breast cancer neoadjuvant chemotherapy in the neo-tango trial. Br J Cancer 108:866-72, 2013

3. Ali HR, Irwin M, Morris L, et al: Astronomical algorithms for automated analysis of tissue protein expression in breast cancer. Br J Cancer 108:602-12, 2013

4. Goode A, Gilbert B, Harkes J, et al: OpenSlide: A vendor-neutral software foundation for digital pathology. J Pathol Inform 4:27, 2013

5. Altman DG, McShane LM, Sauerbrei W, et al: Reporting recommendations for tumor marker prognostic studies (REMARK): explanation and elaboration. BMC medicine 10:51, 2012

6. McShane LM, Altman DG, Sauerbrei W, et al: Reporting recommendations for tumor marker prognostic studies (REMARK). J Natl Cancer Inst 97:1180-4, 2005
