# Supplementary figures and images for: Computational pathology of pre-treatment biopsies identifies lymphocyte density as a predictor of response to neoadjuvant chemotherapy in breast cancer
Source: Breast Cancer Res. 2016 Feb 16;18:21. doi: 10.1186/s13058-016-0682-8 (PMC4755003; doi:10.1186/s13058-016-0682-8)

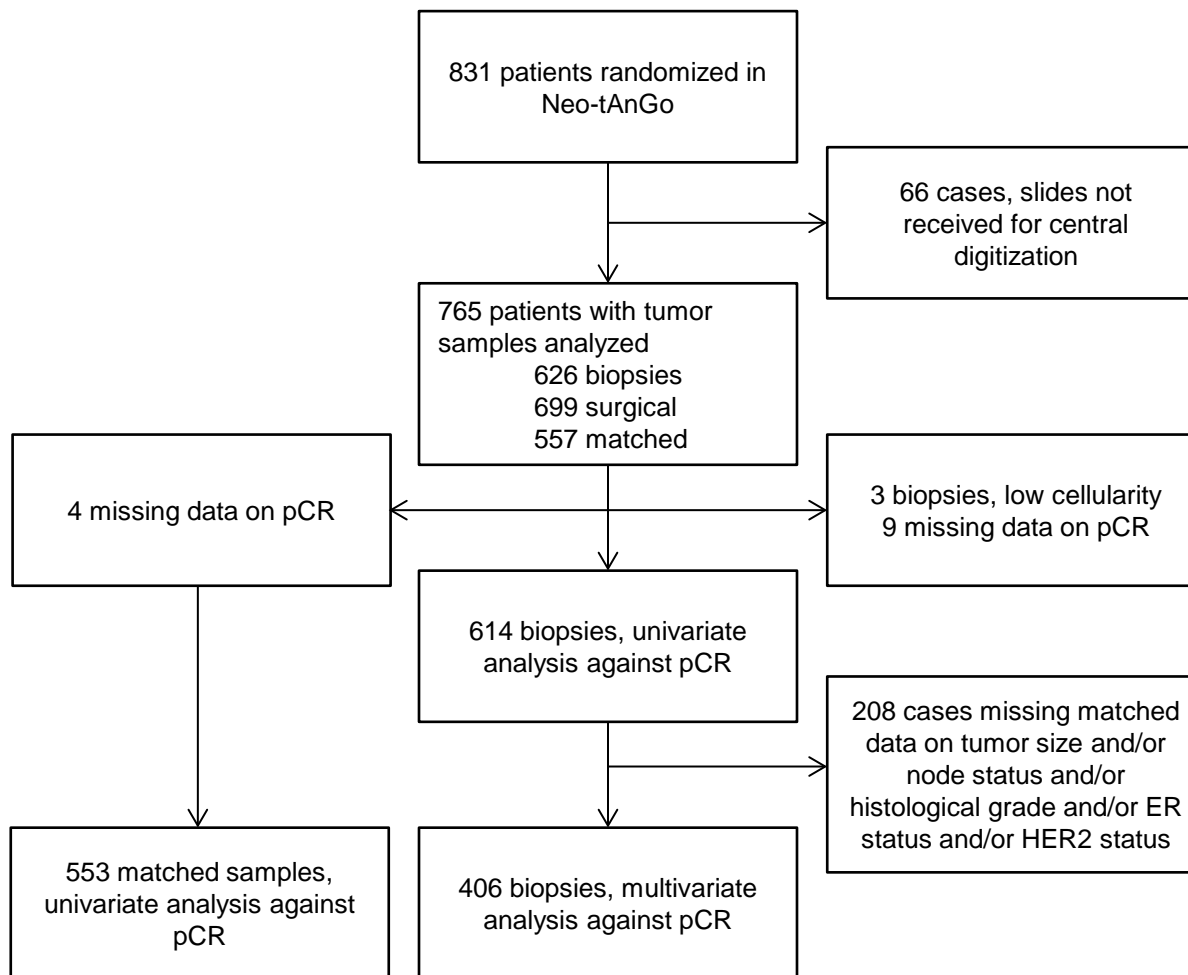

Supplement: Additional file 2: — CONSORT diagram illustrating the flow of patients at each analytical stage. (PDF 7 kb) [file 13058_2016_682_MOESM2_ESM.pdf]

## Cancer

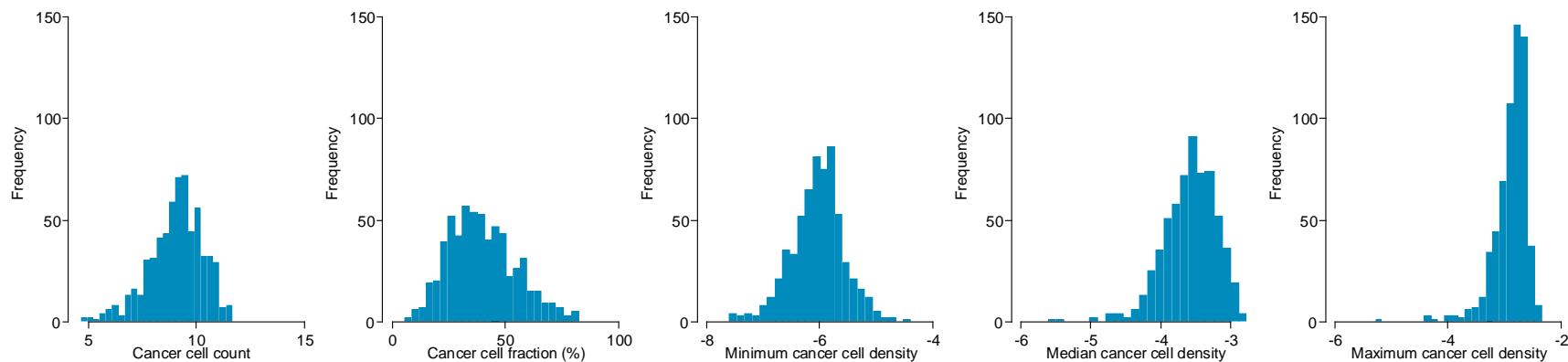

## Stromal

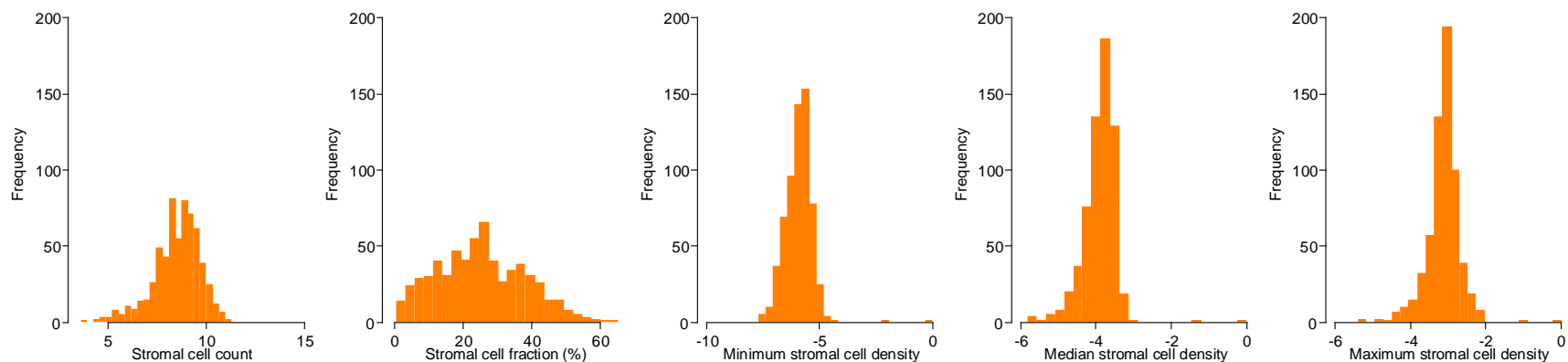

## Lymphocyte

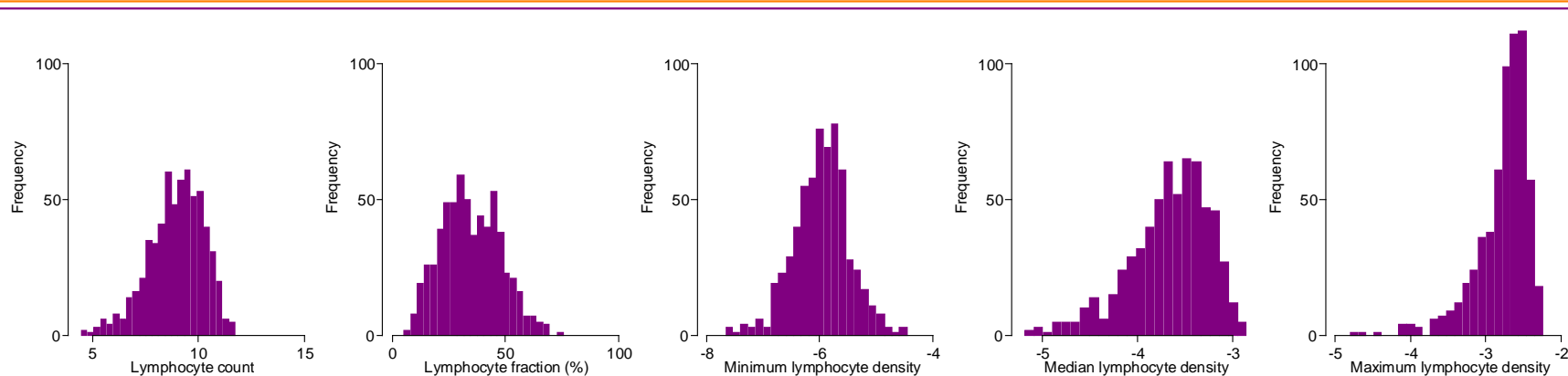

Supplement: Additional file 3: — Histograms depicting the distribution of all image metrics from pre-treatment samples. (PDF 25 kb) [file 13058_2016_682_MOESM3_ESM.pdf]

## Cancer

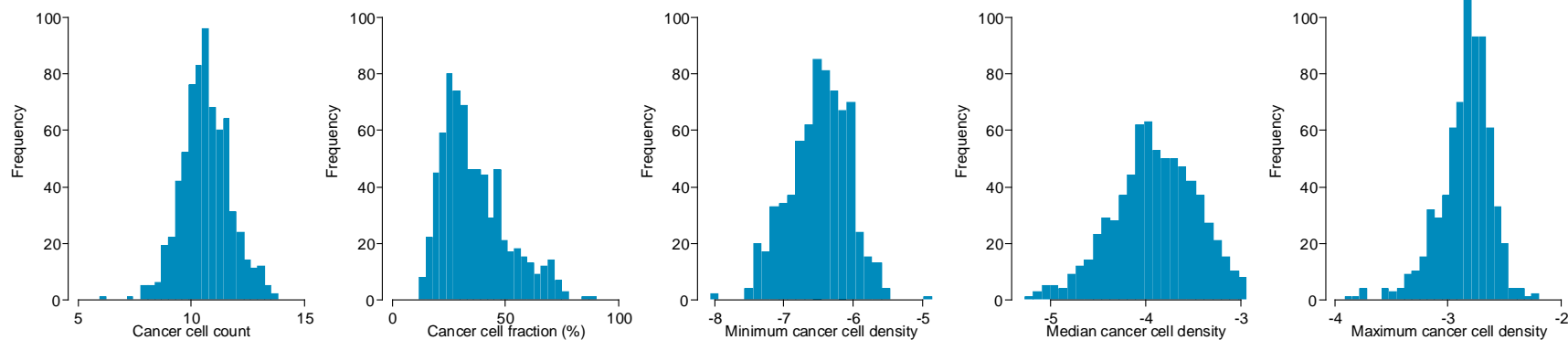

## Stromal

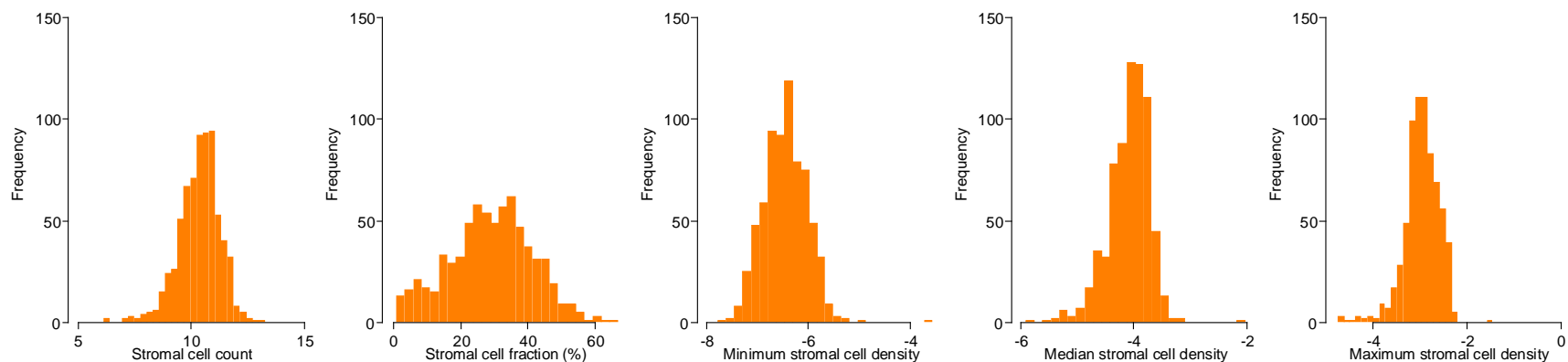

## Lymphocyte

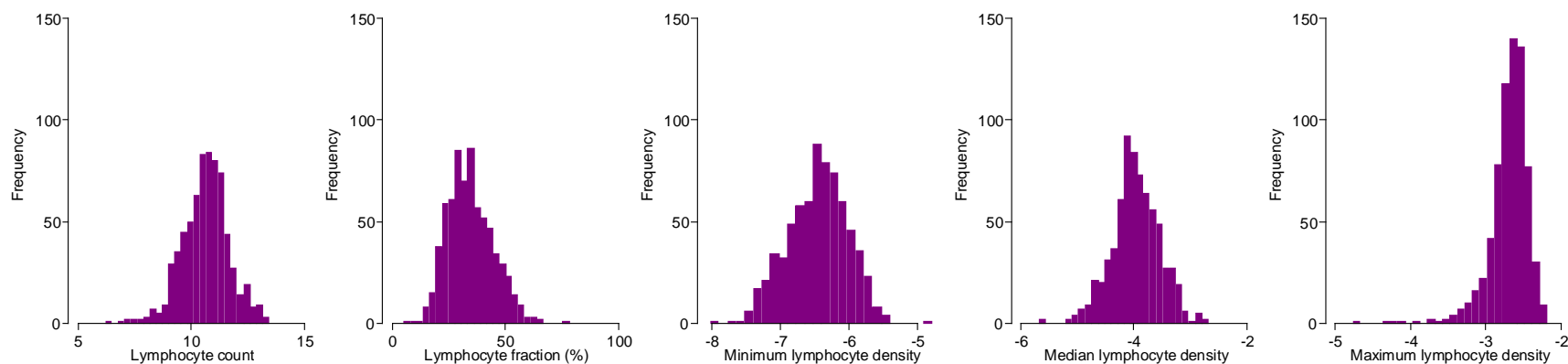

Supplement: Additional file 4: — Histograms depicting the distribution of all image metrics from post-treatment surgical samples. (PDF 26 kb) [file 13058_2016_682_MOESM4_ESM.pdf]

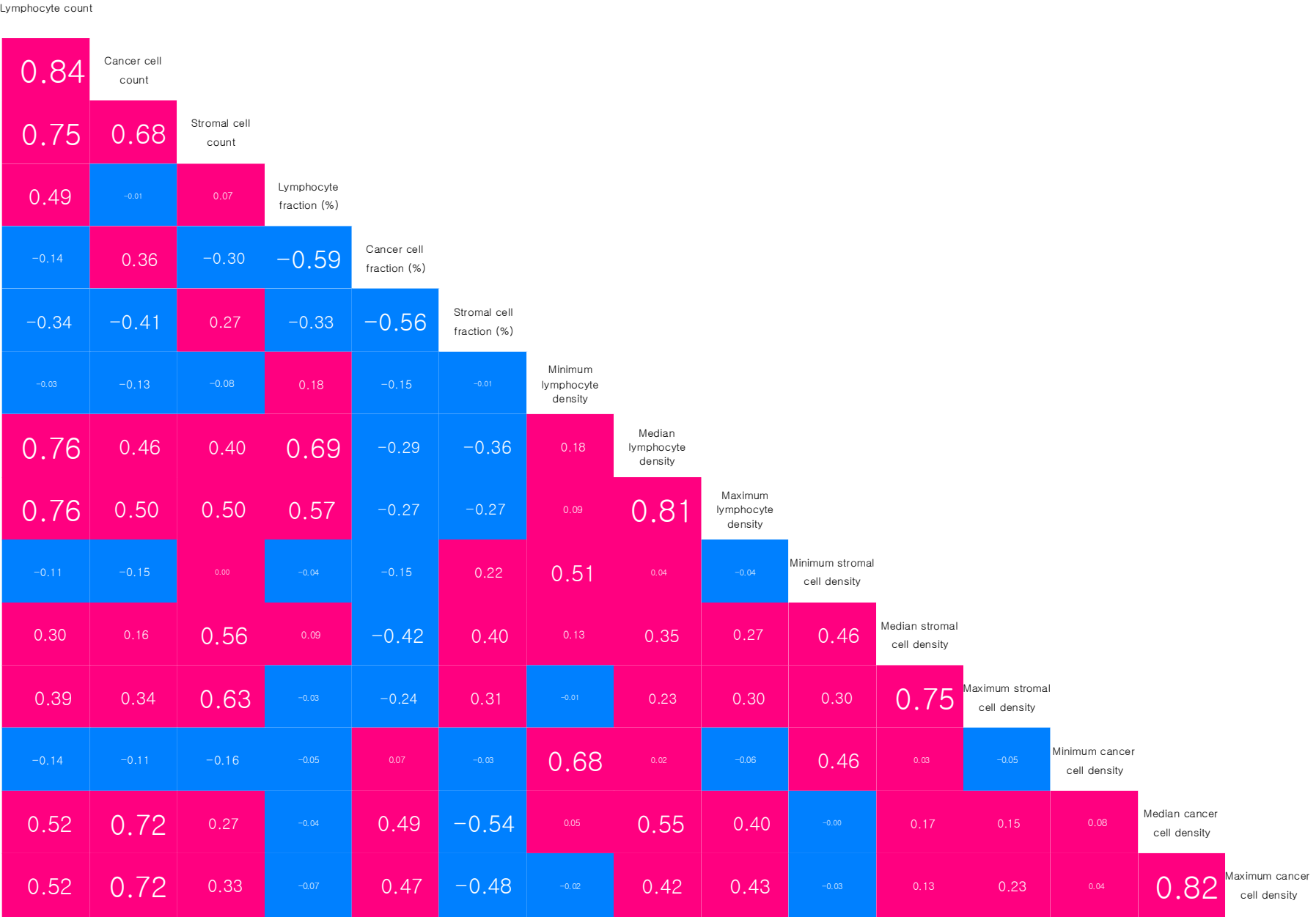

Supplement: Additional file 5: — Correlation matrix of all image metrics from pre-treatment samples. Correlation matrix of 15 image metrics derived from pre-treatment biopsies. Correlations are Pearson’s coefficients. Text size is proportional to the strength of the correlation. Pink boxes denote positive correlations and blue boxes denote negative correlations. (PDF 82 kb) [file 13058_2016_682_MOESM5_ESM.pdf]

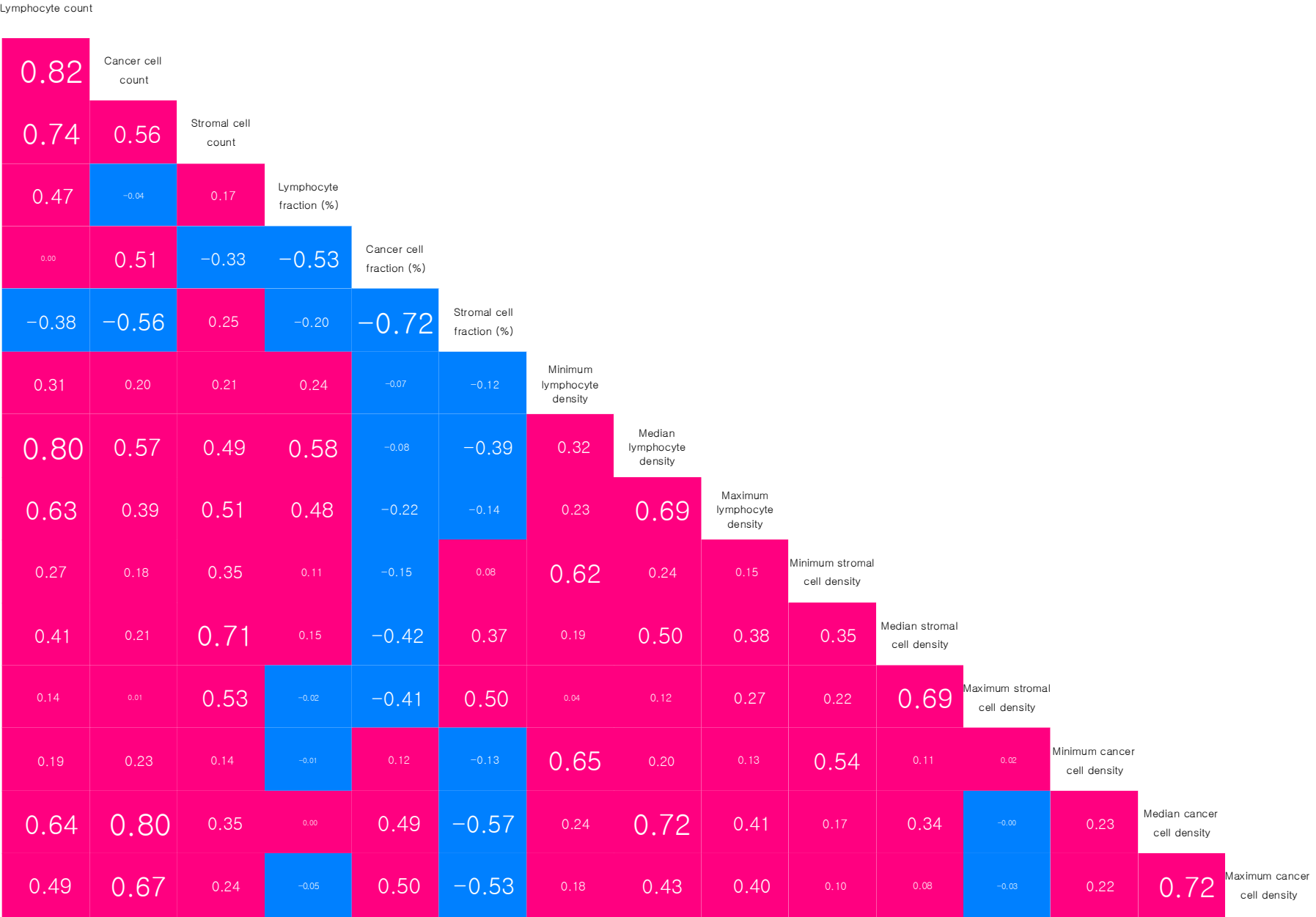

Supplement: Additional file 6: — Correlation matrix of all image metrics from post-treatment surgical samples. Correlation matrix of 15 image metrics derived from post-treatment surgical samples. Correlations are Pearson’s coefficients. Text size is proportional to the strength of the correlation. Pink boxes denote positive correlations and those blue boxes denote negative correlations. (PDF 82 kb) [file 13058_2016_682_MOESM6_ESM.pdf]

## Cancer

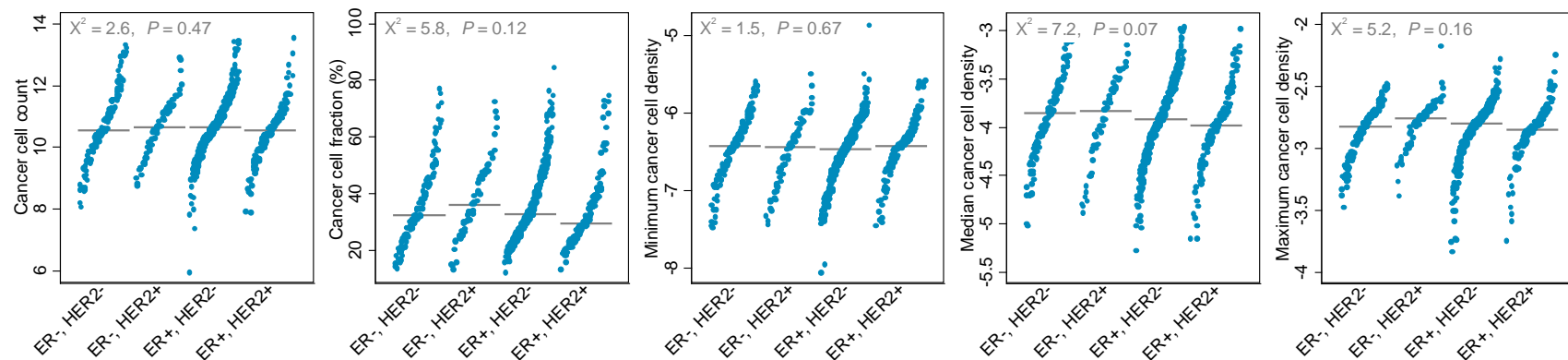

## Stromal

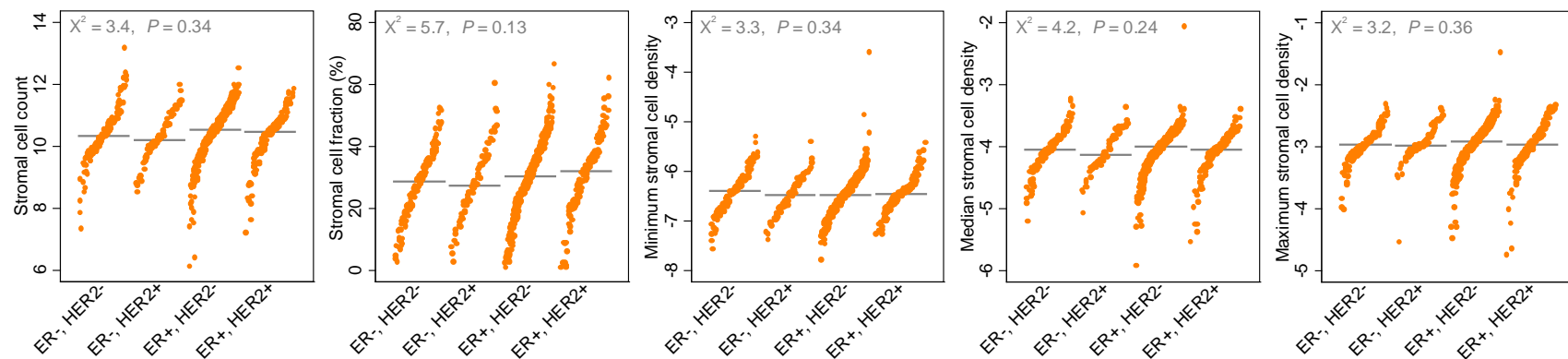

## Lymphocyte

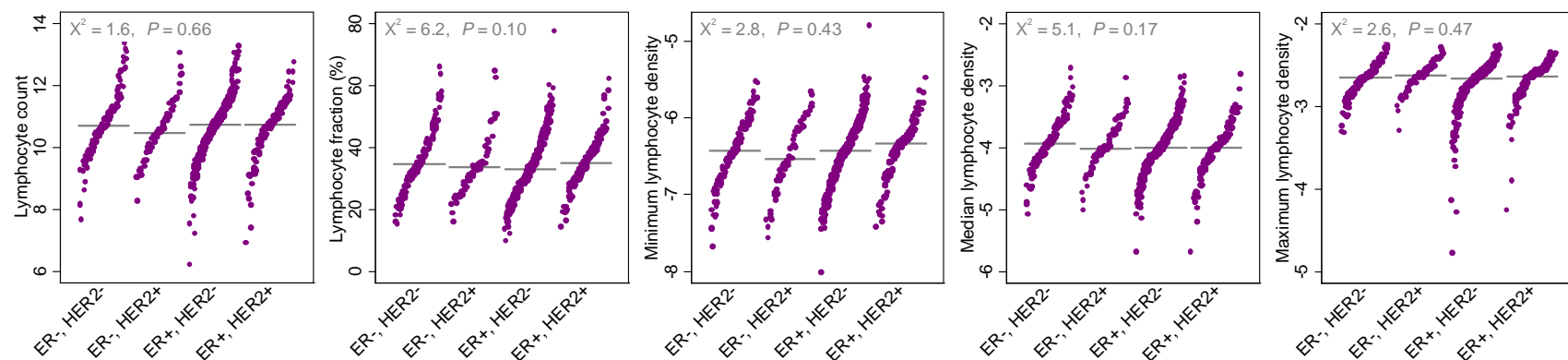

Supplement: Additional file 7: — Distribution of post-treatment sample image metrics by tumor molecular subtype. Horizontal gray lines represent median values. Results of Kruskal-Wallis tests are depicted within graphs; red text denotes p values <0.05. (PDF 177 kb) [file 13058_2016_682_MOESM7_ESM.pdf]

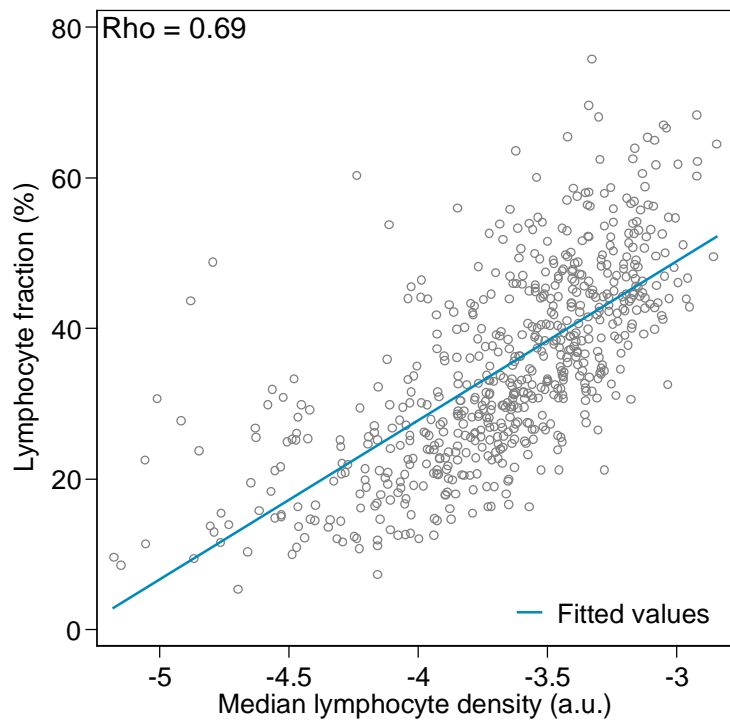

Supplement: Additional file 10: — Scatter plot of median lymphocyte density and lymphocyte fraction in pre-treatment biopsies. Depicted rho value is a Pearson’s correlation coefficient. a.u. arbitrary units. (PDF 19 kb) [file 13058_2016_682_MOESM10_ESM.pdf]

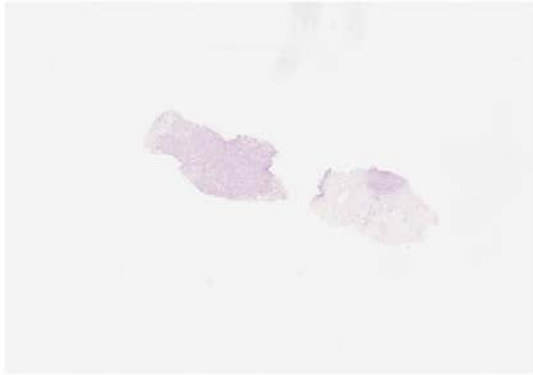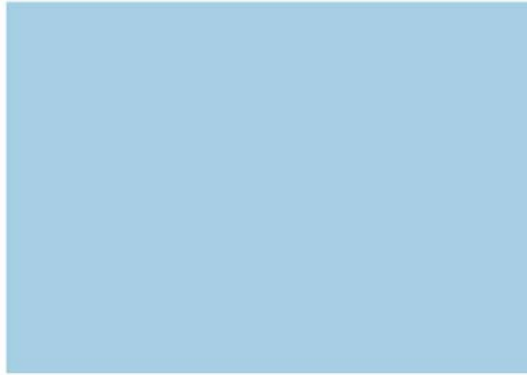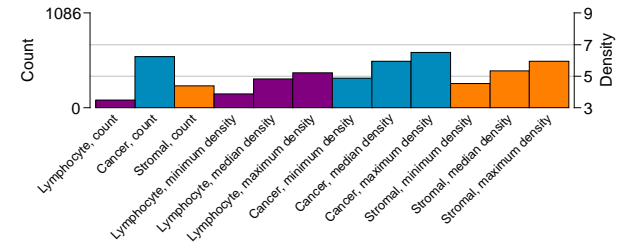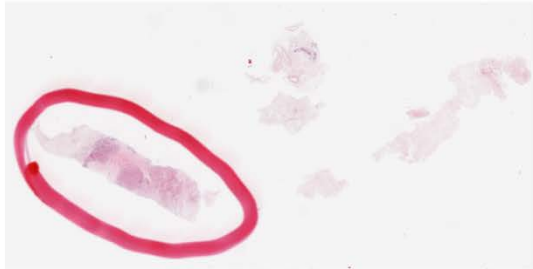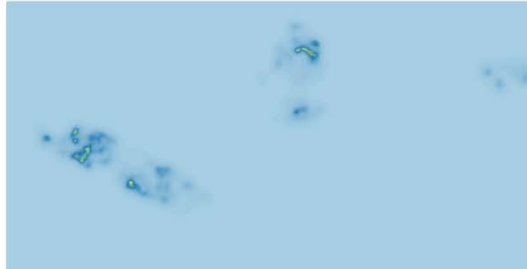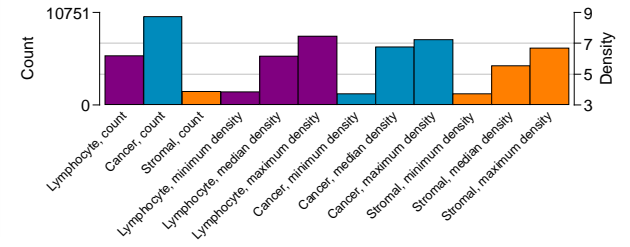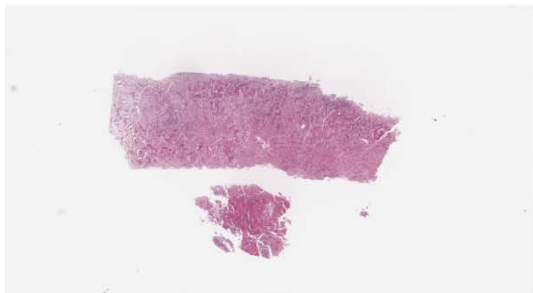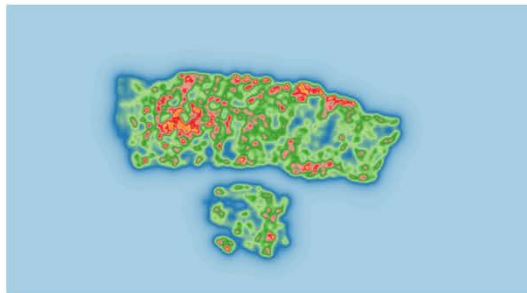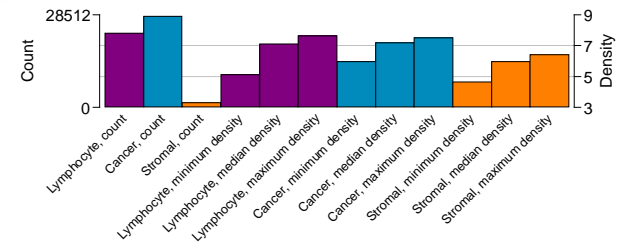

Lymphocyte density

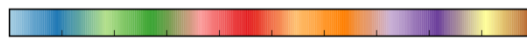

Low

High

Supplement: Additional file 11: — Examples of images and associated metrics from cases with varying median lymphocyte density. Depicted density metrics have been arbitrarily offset to depict positive values but are normalized across samples, hence comparable between plots (right y-axis). Note that absolute counts are depicted against different scales (left y-axis). (PDF 63 kb) [file 13058_2016_682_MOESM11_ESM.pdf]

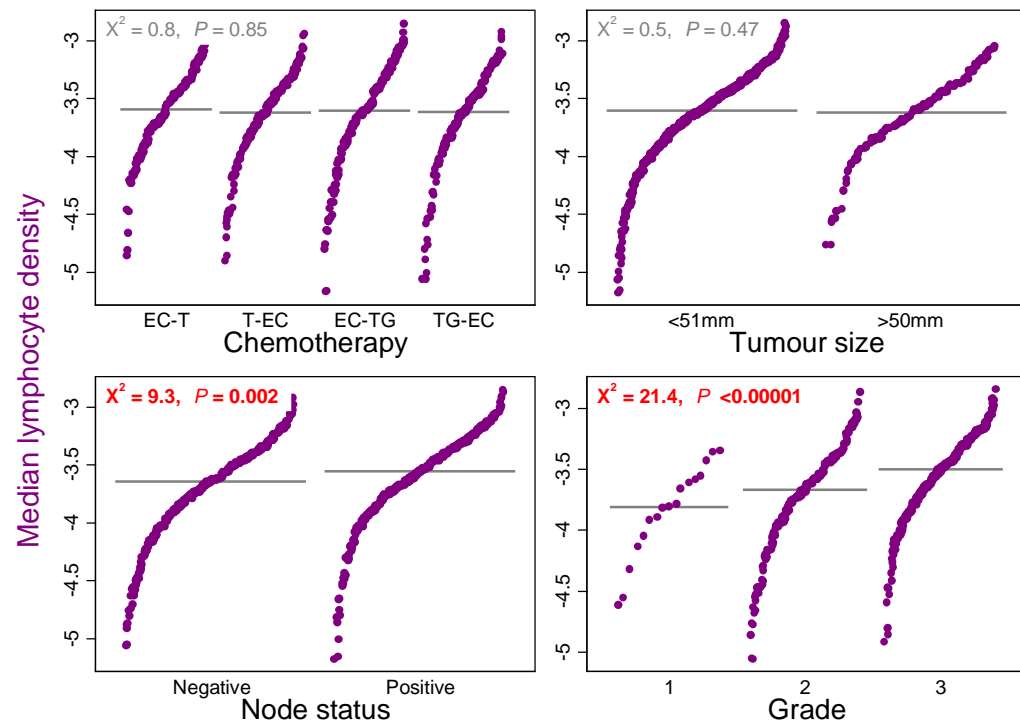

Supplement: Additional file 12: — Distribution of median lymphocyte density from pre-treatment biopsies by clinical variables. Horizontal gray lines represent median values. Results of Kruskal-Wallis tests are depicted within graphs; red text denotes p values <0.05. (PDF 62 kb) [file 13058_2016_682_MOESM12_ESM.pdf]
