# Supplementary material for: Computational pathology of pre-treatment biopsies identifies lymphocyte density as a predictor of response to neoadjuvant chemotherapy in breast cancer
Source: Breast Cancer Res. 2016 Feb 16;18:21. doi: 10.1186/s13058-016-0682-8 (PMC4755003; doi:10.1186/s13058-016-0682-8)
Supplement: Additional file 8: — Univariate and multivariate logistic regression models for all 15 image metrics. (DOCX 16 kb) [file 13058_2016_682_MOESM8_ESM.docx]

**Additional File 8.** Univariate and multivariate logistic regression models for all fifteen image metrics.

|  |  | **Univariate** | | | |  | **Multivariate** | | | |
| --- | --- | --- | --- | --- | --- | --- | --- | --- | --- | --- |
| **Variable** | **Categories** | **Odds ratio** | **95% CI** | **P-value** | **Observations** |  | **Odds ratio** | **95% CI** | **P-value** | **Observations** |
| Cancer cell count | Continuous | 1.10 | 0.92-1.31 | 0.31 | 614 |  | 2.17 | 1.21-3.87 | 0.009 | 614 |
| Stromal cell count | Continuous | 1.05 | 0.87-1.26 | 0.62 | 614 |  |  |  |  |  |
| Lymphocyte count | Continuous | 1.22 | 1.02-1.46 | 0.03 | 614 |  | 0.39 | 0.22-0.71 | 0.002 | 614 |
| Cancer cell fraction (%) | Continuous | 0.99 | 0.98-1.01 | 0.29 | 614 |  |  |  |  |  |
| Stromal cell fraction (%) | Continuous | 0.99 | 0.97-1.00 | 0.11 | 614 |  |  |  |  |  |
| Lymphocyte fraction (%) | Continuous | 1.02 | 1.01-1.04 | 0.006 | 614 |  |  |  |  |  |
| Minimum cancer cell density | Continuous | 0.71 | 0.45-1.13 | 0.15 | 614 |  | 0.59 | 0.37-0.96 | 0.04 | 614 |
| Maximum cancer cell density | Continuous | 1.12 | 0.54-2.36 | 0.76 | 614 |  | 0.17 | 0.045-0.64 | 0.009 | 614 |
| Median cancer cell density | Continuous | 2.03 | 1.08-3.79 | 0.03 | 614 |  |  |  |  |  |
| Minimum stromal cell density | Continuous | 1.13 | 0.80-1.59 | 0.49 | 614 |  |  |  |  |  |
| Maximum stromal cell density | Continuous | 1.42 | 0.87-2.32 | 0.16 | 614 |  |  |  |  |  |
| Median stromal cell density | Continuous | 1.65 | 1.00-2.70 | 0.05 | 614 |  |  |  |  |  |
| Minimum lymphocyte density | Continuous | 0.88 | 0.57-1.36 | 0.56 | 614 |  |  |  |  |  |
| Maximum lymphocyte density | Continuous | 3.48 | 1.54-7.86 | 0.003 | 614 |  |  |  |  |  |
| Median lymphocyte density | Continuous | 4.46 | 2.34-8.50 | <0.00001 | 614 |  | 24.8 | 7.50-82.0 | <0.00001 | 614 |
