# Supplementary material for: Computational pathology of pre-treatment biopsies identifies lymphocyte density as a predictor of response to neoadjuvant chemotherapy in breast cancer
Source: Breast Cancer Res. 2016 Feb 16;18:21. doi: 10.1186/s13058-016-0682-8 (PMC4755003; doi:10.1186/s13058-016-0682-8)
Supplement: Additional file 9: — Multivariate logistic regression model including clinical prognostic factors and image metrics derived from model depicted in Additional File 8. (DOCX 14 kb) [file 13058_2016_682_MOESM9_ESM.docx]

**Additional File 9.** Multivariate logistic regression model including clinical prognostic factors and image metrics derived from model depicted in Table S1.

|  |  | **Multivariate** | | | |
| --- | --- | --- | --- | --- | --- |
| **Variable** | **Categories** | **Odds ratio** | **95% CI** | **P-value** | **Observations** |
| Cancer cell count | Continuous | 1.38 | 0.64-2.98 | 0.41 | 406 |
| Minimum cancer cell density | Continuous | 0.62 | 0.33-1.16 | 0.13 | 406 |
| Lymphocyte count | Continuous | 0.73 | 0.34-1.59 | 0.43 | 406 |
| Median lymphocyte density | Continuous | 4.96 | 1.06-23.2 | 0.04 | 406 |
| Maximum cancer cell density | Continuous | 0.29 | 0.054-1.59 | 0.15 | 406 |
| Tumour size | ≤50mm, >50mm | 0.75 | 0.28-1.98 | 0.56 | 406 |
| Node status | Negative, Positive | 0.53 | 0.29-0.98 | 0.04 | 406 |
| Grade | 1,2,3 | 3.46 | 1.64-7.31 | 0.001 | 406 |
| ER status | Negative, Positive | 0.29 | 0.16-0.53 | 0.00005 | 406 |
| HER2 status | Negative, Positive | 1.81 | 0.97-3.39 | 0.06 | 406 |
